# Biochemical, Physiological and Anatomical Mechanisms of Adaptation of *Callistemon citrinus* and *Viburnum lucidum* to NaCl and CaCl_2_ Salinization

**DOI:** 10.3389/fpls.2019.00742

**Published:** 2019-06-04

**Authors:** Chiara Cirillo, Veronica De Micco, Carmen Arena, Petronia Carillo, Antonio Pannico, Stefania De Pascale, Youssef Rouphael

**Affiliations:** ^1^Department of Agricultural Sciences, University of Naples Federico II, Naples, Italy; ^2^Department of Biology, University of Naples Federico II, Naples, Italy; ^3^Department of Environmental, Biological and Pharmaceutical Sciences and Technologies, University of Campania “Luigi Vanvitelli”, Caserta, Italy

**Keywords:** arrow-wood, chlorophyll fluorescence, functional leaf anatomical traits, gas exchanges, osmoregulation, red bottlebrush, SPAD index, water relations

## Abstract

*Callistemon citrinus* and *Viburnum lucidum* are appreciated and widespread ornamental shrubs for their abundant flowering and/or brilliant foliage. The intrinsic tolerance to drought/salinity supports their use in urban areas and in xeriscaping. Despite adaptive responses of these ornamental species to sodium chloride (NaCl) have been extensively explored, little is known on the effects of other salt solution, yet iso-osmotic, on their growth, mineral composition and metabolism. The present research was aimed to assess responses at the biochemical, physiological and anatomical levels to iso-osmotic salt solutions of NaCl and CaCl_2_ to discriminate the effects of osmotic stress and ion toxicity. The two ornamental species developed different salt-tolerance mechanisms depending on the salinity sources. The growth parameters and biomass production decreased under salinization in both ornamental species, independently of the type of salt, with a detrimental effect of CaCl_2_ on *C. citrinus.* The adaptive mechanisms adopted by the two ornamental species to counteract the NaCl salinity were similar, and the decline in growth was mostly related to stomatal limitations of net CO_2_ assimilation rate, together with the reduction in leaf chlorophyll content (SPAD index). The stronger reduction of *C. citrinus* growth compared to *V. lucidum*, was due to an exacerbated reduction in net photosynthetic rate, driven by both stomatal and non stomatal limitations. In similar conditions, *V. lucidum* exhibited other additional adaptive response, such as modification in leaf functional anatomical traits, mostly related to the reduction in the stomata size allowing plants a better control of stomata opening than in *C. citrinus*. However, *C. citrinus* plants displayed an increased ability to retain higher Cl^-^ levels in leaves than in roots under CaCl_2_ salinity compared to *V. lucidum*, thus, indicating a further attempt to counteract chloride toxicity through an increased vacuolar compartmentalization and to take advantages of them as chip osmotica.

## Introduction

The safeguard of water resources is an important issue for all Mediterranean countries, where the balance between the water demand for irrigation of food crops and its real availability has reached a critical level due to prolonged periods of water shortage and increased frequency of drought events (Ferchichi et al., 2018). The drought, salinity, and pollution from leachate, that contaminates the limited water resources, can have synergistic effects, thus increasing the seriousness of the phenomenon. Nonetheless, in this area highly intensive field and greenhouse cultivation is carried on to meet food demand; while, at the same time, the incessant growth of population is inevitably increasing the problem of water shortage and water quality crises (Saggaï et al., 2017).

Excessive concentrations of sodium chloride (NaCl) can affect and damage plants depending on the sensitivity of the species and/or genotypes (Colla et al., 2012; Liu et al., 2017), the phenological stage (Woodrow et al., 2017), and eventually the crop management practices (Colla et al., 2010). When sensitive plants are exposed to salinity, they first experiment osmotic stress because the increased osmotic strength of the soil solution reduces water uptake, causing inhibition of cell division and enlargement, and limitations of stomatal conductance, transpiration and photosynthesis (Lucini et al., 2015, 2016; Rouphael et al., 2016, 2018). Afterward, during long term salinity, plants withstand ionic stress, responsible for nutritional imbalance, oxidative stress, and inhibition of protein synthesis and enzyme activity, ultimately reducing plant growth, development and survival (Munns and Tester, 2008; Borgognone et al., 2016; Fascella et al., 2017; Carillo, 2018).

The majority of studies carried out on salinity, alone or in combination with other stresses, have mostly used NaCl as principal salt source, and the stress symptoms have been related to Na^+^ toxicity (Annunziata et al., 2017; Rouphael et al., 2017a,b; Woodrow et al., 2017). Less studies have concerned chloride (Geilfus, 2018) and other elements specific toxicity and/or conflicting effects on plants (Colla et al., 2012, 2013; Ntatsi et al., 2017; Rabhi et al., 2018), despite the fact that a multitude of salts are responsible for salinity and some of them, like Na_2_SO_4_ and CaCl_2_, have a concentration even higher than that of NaCl in soil and in groundwater of many areas in the world (Nedjimi et al., 2006; Ezlit et al., 2010). Depending on the type and concentration of salts in the water or growing substrate, ions can exert a beneficial or toxic action, because of a competition effect among ions or predominance of specific ions (Scagel et al., 2017). In particular, Ca^2+^ is able to mitigate salt effects in plants treated with NaCl by improving Ca^2+^/Na^+^ selectivity, and maintaining the structural and functional integrity of the membranes, protecting them from the detrimental effects of Na^+^, as well as reducing the leakage of cytosolic K^+^ (Cramer et al., 1985; Grattan and Grieve, 1999; Renault and Affifi, 2009; Korkmaz et al., 2017). Under short term NaCl stress, cytosolic Ca^2+^ at micromolar concentrations has been proven to bind and open the *Arabidopsis* vacuolar Two Pore K^+^ channel 1 (TPK1) to release K^+^ and maintain favorable Na^+^/K^+^ ratios; while in prolonged stress, sub-micromolar concentration of Ca^2+^ are part of longer-term K^+^ homeostasis in adapted roots (Latz et al., 2013; Wilkins et al., 2016). Addition of only 2–5 mM Ca^2+^ can increase nitrogen, Ca^2+^ and K^+^ selectivity, improve Reactive Oxygen Species (ROS) detoxification and Relative Water Content (RWC), and decrease membrane damages in both shoots and roots of plants under 50–200 mM of NaCl (Parvin et al., 2019).

However, the effectiveness of these responses depends on the source of Ca^2+^ ions (CaCl_2_ or CaSO_4_), in addition to the plant species/genotype reactivity to it (Volkmar et al., 1998). Several studies demonstrated that mild to moderate concentrations of CaCl_2_ can cause osmotic and ion specific effects more phytotoxic than NaCl in different horticultural species (Borghesi et al., 2013; Colla et al., 2013; Borgognone et al., 2014). The reduction in growth and yield observed in plants treated with CaCl_2_ could be attributed to the toxic effects of Cl^-^, whose uptake and transport to leaves seem to be less controlled than that of Na^+^, thus negatively affecting plant metabolism and development (Colla et al., 2013 and references therein). It is well established that herbaceous perennial species can be more sensitive to Cl^-^ than to Na^+^, with critical levels of Cl^-^ varying between 4 and 7 mg g^-1^ (Ju et al., 2018). Chloride toxic effects concern chlorophyll degradation, decline in PSII quantum yield and photosynthetic electron transport rate (Tavakkoli et al., 2010, 2011).

Notwithstanding the sub-optimal characteristics of reclaimed water, in particular for the high CaCl_2_ content, the scarcity of good quality water forces its use as an alternative water supply especially for irrigation. To this aim, being characterized by high adaptability to salinity, ornamental shrub and bush species could provide an interesting model to study the effects of salty wastewater use in plants (Acosta-Motos et al., 2015). Ornamental shrubs, naturally present in coastal arid and semiarid areas, as well as in marginal soils, are, in fact, endowed with unique morpho-anatomical, physiological and biochemical traits which allow them to cope with salinity, while maintaining good growth rate and abundant flowering, and therefore preserving their ornamental value (Tattini et al., 2006; Carillo et al., 2019).

*Callistemon citrinus* (Myrtaceae), known as red bottlebrush, and *Viburnum lucidum* (Adoxaceae), known as arrow-wood, are of great interest because they are highly sought-after on European markets as ornamental potted shrubs for their abundant flowering with bright colors and peculiar shapes and/or brilliant foliage (Vernieri et al., 2006; Gori et al., 2008), in addition to their moderate to high tolerance to drought and salinity which makes them suitable for use in urban areas and xeriscaping (Lippi et al., 2003; Vernieri et al., 2006; Álvarez and Sánchez-Blanco, 2014; Cirillo et al., 2014, 2016, 2017). Notwithstanding several studies have been performed to assess the physiological responses of these ornamental species to NaCl (Lippi et al., 2003; Álvarez and Sánchez-Blanco, 2015; Cirillo et al., 2016), little is known on the effects of other types of salinity on their growth, mineral composition and metabolism. Indeed, it is difficult to discriminate among plant responses due to osmotic and ionic stresses. Since osmotic stress is always proportional to salt concentration, iso-osmotic solutions of different salt types may trigger the same plant growth and physiological responses due to osmotic unbalance, still causing ion-specific stress effects linked to salt type and genotype-specific sensitivity (Pagter et al., 2009). Thus, it is of great importance to elucidate the biochemical, physiological and anatomical mechanisms of adaptation of potted *C. citrinus* and *V. lucidum* to iso-osmotic salt solutions of NaCl and CaCl_2_ in order to discriminate the effects of osmotic stress and ion toxicity, but excluding the further problem of Ca^2+^ deficiency. The responses of the two species to the two chloride salts were analyzed in terms of growth parameters, leaf gas exchange, photosystem efficiency, water relations and leaf functional anatomical traits. To achieve a comprehensive and integrated interpretation of all data, we applied tools of multivariate statistics, whose usefulness has been recently proved in horticultural species, including ornamentals, in response to salinity concentration (Rouphael et al., 2018; Carillo et al., 2019). The knowledge gained from this study could be used as a meaningful approach to discriminate between osmotic and ionic stresses responses in plants under salt stress conditions, in addition to provide practical specialist skills for the alternative re-use of reclaimed water for xeriscaping of urban, industrial and marginal areas of Mediterranean countries.

## Materials and Methods

### Plant Material and Experimental Conditions

Rooted cuttings of 2-year-old *Callistemon citrinus and Viburnum lucidum*, purchased from a specialized nursery (Vivaio Torsanlorenzo, Ardea, Italy), were transplanted on March 12 2014 into 1.5 L plastic pots filled with peat moss. Plants were placed inside a zinc-coated steel greenhouse at the experimental station of the University of Naples Federico II, South Italy (43° 31′ N, 14° 58′ E; 60 m above sea level). The pots were placed on 1.8 m wide × 7 m-long troughs, at a plant density of 2.4 per square meter. Plants were cultivated under natural light conditions using a 50% black shading net and photosynthetically active radiation (PAR) averaged 550 μmol m^-2^ s^-1^. Inside the glasshouse, the daily air temperature varied between 17 and 34°C and the relative humidity (R.H.) was 58%/75% during day/night, respectively.

For both ornamental species, the experiment treatments consisted of three nutrient solutions, a basic nutrient solution (control non-salt treatment) and two saline nutrient solutions obtained by adding 80 mM NaCl or 53.3 mM CaCl_2_ to the control solution. The study was performed in terms of equimolar concentrations of the two different chloride salts, in order to assess the salt-specific influence. Treatments were arranged in a completely randomized blocks design (CRBD) with four replicates per treatment. Each experimental unit included six plants (*n* = 72 plants for each species). The first and last plant of each experimental unit were considered as guards.

### Nutrient Solution Management, Sodium Chloride and Calcium Chloride Application

The quality of the irrigation water was typical of the area and characterized by a high bicarbonate concentration (5.5 mM HCO_3_), 0.5 mM Ca, 0.5 mM Mg, 0.1 mM K, 0.4 mM Na, and 0.3 mM Cl. Values of the pH and EC of the irrigation water were 7.0 and 0.4 dS m^-1^, respectively. The composition of the basic nutrient solution (non-salt control) used to replenish nutrients absorbed by plants was as follows: 13.6 mM N-NO_3_, 2.0 mM S,1.4 mM P, 6.0 mM K, 4.5 mM Ca, 2.0 mM Mg, 1 mM Na, 1 mM Cl, 20 μM Fe, 9 μM Mn, 1.5 μM Cu, 3 μM Zn, 20 μM B and 0.3 μM Mo (Carillo et al., 2019) with an electrical conductivity (EC) of 2.0 dS m^-1^. The irrigation water 0.5 mM Ca natural content, and the additional 4.5 mM Ca in the basic nutrient solution, allowed to have a basic nutrient media containing 5 mM Ca, to allow plants having an optimal Ca concentration (Parvin et al., 2019). The saline nutrient solutions had the same basic composition, plus an additional 80 mM NaCl or 53.33 mM CaCl_2_, resulting in an EC values of 11.1 and 11.6 dS m^-1^, respectively. The pH in the three nutrient solutions was 6.0 ± 0.3. Saline treatments started on March 25 (12 days after transplanting, 12 DAT).

The nutrient solutions in all treatments were pumped from independent tanks through a drip irrigation system, with two emitters per plant at a flow rate of 2 L h^-1^ each. Irrigation timing varied from 2 to 4 fertigations per day of 1–3 min. The watering was performed with a leaching fraction of 25% higher than the amount of water required to restore field capacity. Leaching fractions of 20-130% are needed to maintain the EC in the growing medium at recommended level (Carillo et al., 2019).

### Biomass Production, Partitioning and Growth Analysis

For both *C. citrinus* and *V. lucidum*, ten plants were sampled to measure the fresh and dry weight of the plants at the beginning of the experiment. At the end of the trial (127 DAT), four plants per experimental unit were harvested and dissected into leaves, stems and roots. The total leaf area per plant was measured using an electronic area meter (Li-Cor3000, Li-Cor, Lincoln, NE, United States), and the number of leaves as well as the plant height were also recorded. Then, all plant organs were dried to constant weight in a forced-air oven at 80°C for 72 h to determine the dry biomass. Shoot dry weight was equal to the sum of the aerial vegetative parts (leaves + stems), and the root-to-shoot ratio was also calculated. Dried plant organs were then sampled for ion analyses. Lastly, the relative growth rate (g g^-1^ day^-1^) was calculated using the equation reported by De Groot et al. (2001): RGR = (lnW_2_-lnW_1_)/(t_2_-t_1_) where W_1_ and W_2_ are the fresh masses (g) of the above-ground plant part (shoot) at times t_1_ and t_2_, corresponding to the beginning (1 DAT) and to the end of the experiment (127 DAT), respectively.

### Soil Plant Analysis Development Index, Leaf Gas Exchange and Chlorophyll *a* Fluorescence Emission

At 122 DAT, the Soil Plant Analysis Development (SPAD) index was measured on fully expanded leaves of *C. citrinus* and *V. lucidum* by means of a portable chlorophyll meter SPAD-502 (KonicaMinolta, Japan) of five representative plants per experimental unit. Measurements were made by avoiding major veins, leaflet margins and damaged areas. Twenty leaves were randomly measured and averaged to a single SPAD value per each replicate.

On the same date, measurements of leaf gas exchanges and chlorophyll *a* fluorescence emission were conducted within 2 h across solar noon (i.e., between 11:00 and 13:00 h) on the youngest fully expanded leaves, using six replicates per each treatment as described in Carillo et al. (2019). Briefly, the net CO_2_ assimilation rate (P_n_), stomatal conductance (g_s_) and transpiration rate (E) were determined with a portable gas-exchange analyzer (LCA 4; ADC BioScientific Ltd., Hoddesdon, United Kingdom), equipped with a broad-leaf PLC (cuvette window area, 6.25 cm^2^). PAR, R.H., and carbon dioxide concentrations were set at ambient value and the flow rate of air was 400 ml s^-1^.

Fluorescence measurements were also performed on six replicates per each treatment. For the chlorophyll *a* fluorescence emission measurements, a portable FluorPen FP100max fluorometer, equipped with a light sensor (Photon System Instruments, Brno, Czechia) was used. The ground fluorescence signal, F_o_, was induced on 30′ dark adapted leaves, by a blue LED internal light of about 1–2 μmol m^-2^ s^-1^. The maximal fluorescence level in the dark, F_m_, was induced by a 1s saturating light pulse of 3000 μmol m^-2^ s^-1^. The maximum quantum efficiency of PSII photochemistry, F_v_/F_m_, was calculated as (F_m_ - F_o_)/F_m_, according to Kitajima and Butler (1975). For the fluorescence measurements in the light, the fluorometer FluorPen FP100max was equipped with an open leaf-clip suitable for measurements under ambient light. The quantum yield of PSII electron transport (QY) was determined according to Genty et al. (1989). The linear electron transport rate (ETR) was expressed following Krall and Edwards (1992), whereas the photochemical (qP) and non-photochemical quenching (NPQ) were calculated as described by Quick and Horton (1984) and Bilger and Björkman (1990), respectively.

### Stem Water Potential

Stem water potential (Ψ_mds_) was measured at the same date of the physiological and biochemical measurements (122 DAT) by selecting one well-lit leaf per plant on three plants per replication. Stem water potential was measured using the pressure chamber (3005-series portable plant water status console, Soil Moisture Equipment Corp., Santa Barbara, CA, United States) technique (Scholander et al., 1965), taking the precautions proposed by Turner and Long (1980). The stem water potential measurement was made at midday (i.e., 12:00 h) on leaves located close to the trunk, bagged for at least 1 h before measurement.

### Leaf and Root Mineral Analysis

The leaf and root dry tissues were finely ground through a mill (IKA, MF10.1, Staufen, Germany) with 0.5 mm-sieve, then 1 g samples were used for macro-minerals, sodium and chloride analyses. Nitrogen (total *N*) concentration in leaf and root tissues was determined after mineralization with sulfuric acid in the presence of potassium sulfate and a low concentration of copper by the Kjeldahl method (Bremner, 1965).

For the anions (PO_4_^3-^, SO_4_^2-^ and Cl^-^) and cations (K^+^, Ca^2+^, Mg^2+^ and Na^+^) leaf and root analysis, 250 mg of the *C. citrinus* and *V. lucidum* dried material were suspended in 50 ml of ultrapure water (Milli-Q, Merck Millipore, Darmstadt, Germany) and subjected to three freeze-thaw cycles in liquid nitrogen followed by 10 min shaking in a water bath (ShakeTemp SW22, Julabo, Seelbach, Germany) at 80°C. The mixture was centrifuged at 6000 rpm for 10 min (R-10 M, Remi Elektrotechnik Limited, India), then filtered through a 0.20 μm filter paper (Whatman International Ltd., Maidstone, United Kingdom), as described previously in Rouphael et al. (2017c). Anions and cations in both *C. citrinus and V. lucidum* organs were separated and quantified by ion chromatography (ICS-3000, Dionex, Sunnyvale, CA, United States) coupled to a conductivity detector. The conductivity detector with IonPac CG12A (4 × 250 mm, Dionex, Corporation) guard column and IonPac CS12A (4 × 250 mm, Dionex, Corporation) analytical column were used for the cations analysis, while for the anions determination, an IonPac AG11-HC guard (4 × 50 mm) column and IonPac AS11-HC analytical column (4 × 250 mm) were employed.

### Analysis of Leaf Anatomical Traits

On the same date of the physiological and biochemical measurements (122 DAT), leaves of both *C. citrinus* and *V. lucidum* were sampled by cutting three fully expanded leaves from three plants per treatment, and immediately fixing them in FAA (5 ml 40% formaldehyde, 5 ml glacial acetic acid, 90 ml 50% ethanol). Each leaf was dissected to remove the apical and basal portions, and dividing the median region of the lamina into two sub-samples: one for stomata analysis, the other for mesophyll characterization through thin sectioning.

Stomata characterization was performed on both adaxial and abaxial lamina surfaces in *C. citrinus*, being its leaves bifacial and amphystomatic, while only on the abaxial surface in dorsiventral *V. lucidum* leaves. Regions of the leaf lamina (5 × 5 mm) were dissected and directly mounted on microscope slides with distilled water and kept pressed by sealing the cover slip with adhesive tape to maintain the lamina as flattened as possible. The slides were observed under an epi-fluorescence microscope (BX60, Olympus, Hamburg, Germany) equipped with a Mercury lamp, band-pass filter 330–385 nm, dichromatic mirror 400 nm and above, and barrier filter 420 nm and above. Such filters allow highlighting stomata among epidermal cells, owing to the different excitation and emission properties of various compounds that make visible the guard cells, especially the thickened inner cell wall at the stomata aperture level (Ruzin, 1999; De Micco et al., 2011). Images of the lamina surface, from three separate regions avoiding main veins, were collected by means of a digital camera (CAMEDIA C4040, Olympus), taking care to avoid veins.

The following parameters were measured through the software program AnalySIS 12.0 (Olympus, Germany): stomata frequency (SF, calculated by counting the number of stomata in a given region of the epidermis and expressed as the number of stomata per mm^2^), and guard cell length (GCL, quantified by measuring the length, pole to pole) of 15 cells.

From the second group of subsamples, 5 × 5 mm portions of the leaf lamina were dissected and dehydrated in an ethanol series (up to 95%), infiltrated and embedded in the JB4 acrylic resin (Polysciences, United States). Thin cross sections (5 μm thick) were cut by means of a rotary microtome, stained with 0.025% toluidine blue in 0.1 M citrate buffer at pH 4.0 (Reale et al., 2012), and permanently mounted with mineral oil for microscopy. Sections were analyzed under a transmitted light microscope (BX60, Olympus, Germany) and digital images were collected and analyzed, as reported above, to quantify some functional anatomical traits, including: thickness of leaf lamina (TLL), palisade and spongy parenchymas (TPP and TSP), measured in five positions of the leaf lamina avoiding veins; quantity of intercellular spaces in the spongy parenchyma (ISS - expressed as the percentage of tissue occupied by intercellular spaces over a given surface, in six positions of the leaf lamina; De Micco et al., 2011). ISS was not measured in *C. citrinus* due to the compactness of the mesophyll.

### Statistical Analysis

All experimental data for both ornamental species were statistically analyzed by one-way analysis of variance (ANOVA) using the SPSS 13 software package^1^. To separate treatment means for each measured parameter, Duncan’s Multiple Range Test was performed at a significance level of *p* ≤ 0.05. Principal component analysis (PCA) was also conducted using Minitab 16.2.1 statistical software, aimed to extract trends when multiple qualitative variables were used by formulating new variables correlated to the original ones. The PCA outputs included treatment component scores as well as variable loading to each selected component (Ciarmiello et al., 2015; Rouphael et al., 2017c).

## Results

### Plant Growth and Morphology

The growth parameters (plant height and number of leaves per plant) and biomass production decreased under salinity independently of the type of salt in both ornamental species, with a particular significant detrimental effect of CaCl_2_ on *C. Citrinus* (Table 1). Specifically, in *C. citrinus* the percentage of plant height, shoot dry weight and number of leaves reduction in comparison to non-salinized control plants was 16.1, 16.6, and 23.2% with NaCl and 25.4, 33.2, and 60.7% with CaCl_2_ (Table 1). The lowest biomass production observed in *C. citrinus* plants with CaCl_2_ compared to NaCl treatment was mainly attributed to the reduction in both leaf number and total leaf area (Tables 1, 2). Moreover, in *V. lucidum* the addition of 80 mM NaCl or 53.33 mM CaCl_2_ in the nutrient solution reduced plant height, leaf number per plant, shoot dry biomass as well as the relative growth rate by 24.3, 33.8, 28.1, and 40.0%, respectively, compared to the control with no significant difference between the two salinity sources (Table 1). An opposite trend was observed for the root-to-shoot (R/S); the ratio increased (by 22.4%) from 0.49 to an average of 0.6 in response to nutrient solution salinity (Table 1).

**Table 1:** Effects of salt treatment in the nutrient solution on plant height shoot and root dry weight, root-to-shoot (R/S) ratio, number of leaves and relative growth rate (RGR) of potted *Callistemon citrinus* and *Viburnum lucidum* plants.

	Plant height	Shoot dry weight	Root dry weight		Leaves	RGR
	(cm)	(g plant^-1^)	(g plant^-1^)	R/S	(no. plant^-1^)	(g g^-1^ day^-1^)
***Callistemon citrinus***						
Salt treatment						
Control	112.1 a	269.7 a	101.8	0.37	3929.3 a	0.0053 a
NaCl	94.0 b	224.9 b	96.1	0.43	3018.1 b	0.0042 a
CaCl_2_	83.6 c	179.9 c	86.1	0.48	1544.7 c	0.0026 b
Significance	^∗∗∗^	^∗∗∗^	NS	NS	^∗∗∗^	^∗∗^
***Viburnum lucidum***						
Salt treatment						
Control	67.2 a	214.1 a	106.1	0.49 b	578.3 a	0.0055 a
NaCl	52.0 b	145.9 b	89.9	0.62 a	382.9 b	0.0030 b
CaCl_2_	49.7 b	162.1 b	92.6	0.59 a	383.2 b	0.0036 b
Significance	^∗∗∗^	^∗∗∗^	NS	^∗^	^∗∗∗^	^∗∗∗^

*NS, ^∗^; ^∗∗^, ^∗∗∗^Non significant or significant at *P* < 0.05, 0.01, and 0.001, respectively. Different letters within each column indicate significant differences according to Duncan’s multiple-range test (*P* ≤ 0.05).*

**Table 2:** Effects of salt treatment in the nutrient solution on total leaf area (LA), net photosynthetic rate (P_n_), sub-stomatal CO_2_ concentration (C_i_), stomatal conductance (g_s_), transpiration rate (E), Soil Plant Analysis Development (SPAD) index and midday stem water potential (ψ_mds_) of potted *Callistemon citrinus* and *Viburnum lucidum* plants.

	LA	P_n_	Ci	g_s_	E	SPAD	ψ_mds_
	(m^2^ plant^-1^)	(μmol CO_2_ m^-2^ s^-1^)	(μmol mol^-1^)	(mmol m^-2^ s^-1^)	(mol H_2_O m^-2^ s^-1^)	Index	(MPa)
***Callistemon citrinus***							
Salt treatment							
Control	1.66 a	7.70 a	173.02 b	88.47 a	2.10 a	61.3 a	–0.63 a
NaCl	1.15 b	3.23 b	138.82 b	24.60 b	0.78 b	56.8 b	–1.00 b
CaCl_2_	0.66 c	1.55 c	214.29 a	18.68 b	0.58 b	56.3 b	–1.00 b
Significance	^∗∗∗^	^∗∗∗^	^∗∗^	^∗∗∗^	^∗∗∗^	^∗∗∗^	^∗∗∗^
***Viburnum lucidum***							
Salt treatment							
Control	1.62 a	7.42 a	185.84	98.17 a	2.28 a	63.60 a	– 0.58 a
NaCl	0.80 b	4.51 b	180.13	64.70 b	1.64 b	49.90 b	– 1.00 b
CaCl_2_	0.93 b	3.45 b	184.56	38.46 b	1.21 b	48.80 b	–0.81b
Significance	^∗∗∗^	^∗∗∗^	NS	^∗∗∗^	^∗∗∗^	^∗∗∗^	^∗∗∗^

*NS, ^∗∗^, ^∗∗∗^Non significant or significant at *P* < 0.01, and 0.001, respectively. Different letters within each column indicate significant differences according to Duncan’s multiple-range test (*P* ≤ 0.05).*

### Physiological and Biochemical Parameters

Similarly to the effects on plant growth parameters, the total leaf area and net CO_2_ assimilation rate (P_n_) in *C. citrinus* decreased in response to the increase in salinity concentration in the nutrient solution with detrimental effects more pronounced for CaCl_2_ treatment (Table 2). Furthermore, the addition of 80 mM NaCl or 53.33 mM CaCl_2_ in the nutrient solution reduced stomatal conductance (g_s_) and transpiration rate (E), greenness readings (i.e., SPAD index) and midday stem water potential (ψ_mds_) by 75.5, 67.6, 7.7, and 37.0%, respectively, compared to the control with no significant difference between the two salinity sources (NaCl and CaCl_2_; Table 2). Moreover, it is worth noting that no significant difference among the physiological parameters was recorded in *V. lucidum* plants treated with NaCl or CaCl_2_ (Table 2). Specifically, under both saline conditions, the total leaf area, P_n_, g_s_, E, SPAD index and ψ_mds_ averaged, respectively, 46.6, 46.4, 47.4, 37.7, 22.4, and 55.7% lower than those recorded in non-salinized control plants (Table 2).

The chlorophyll *a* fluorescence analysis evidenced significant differences in photochemistry among salt treatments between *C. citrinus* and *V. lucidum*. More specifically, for both species, the addition of NaCl in the nutrient solution did not determine changes in photochemical quenching (qP), quantum yield of PSII electron transport (QY), maximum quantum efficiency of PSII photochemistry (F_v_/F_m_) and linear electron transport rate (ETR) compared to control (Table 3). Conversely, for both species, the addition of CaCl_2_ in the nutrient solution elicited significant decrease in qP, QY, F_v_/F_m_ and ETR compared to both non-saline and NaCl treatments, whereas an opposite trend was observed for NPQ (Table 3).

**Table 3:** Effects of salt treatment in the nutrient solution on photochemical quenching (qP), quantum yield of PSII electron transport (QY), maximum quantum efficiency of PSII photochemistry (F_v_/F_m_), non-photochemical quenching (NPQ) and linear electron transport rate (ETR) of potted *Callistemon citrinus* and *Viburnum lucidum* plants.

	qP	QY	F_v_/F_m_	NPQ	ETR
***Callistemon citrinus***					
Salt treatment					
Control	0.909 a	0.668 a	0.807 a	0.524 a	169.9 a
NaCl	0.917 a	0.666 a	0.793 a	0.457 a	193.6 a
CaCl_2_	0.885 b	0.527 b	0.707 b	0.700 b	141.2 b
Significance	^∗∗^	^∗∗∗^	^∗∗∗^	^∗∗∗^	^∗∗∗^
***Viburnum lucidum***					
Salt treatment					
Control	0.940 a	0.638 a	0.798 a	0.877 a	188.7 a
NaCl	0.932 a	0.615 a	0.813 a	1.237 b	217.6 a
CaCl_2_	0.900 b	0.538 b	0.760 b	1.428 b	165.5 b
Significance	^∗^	^∗^	^∗^	^∗^	^∗^

*NS, ^∗^; ^∗∗^, ^∗∗∗^Non significant or significant at *P* < 0.05, 0.01, and 0.001, respectively. Different letters within each column indicate significant differences according to Duncan’s multiple-range test (*P* ≤ 0.05).*

### Ions Content and Partitioning

In *C. citrinus*, the total N and PO_4_^3-^ concentrations in leaves were negatively affected by salt stress treatment with more detrimental effects in presence of CaCl_2_ (Table 4). The application of CaCl_2_ in the nutrient solution significantly affected the K^+^ and Mg^2+^ concentrations in leaf tissue as well as Ca^2+^ concentration in both organs, which were higher than in both non-saline and NaCl treatments (Table 4). Sodium (Na^+^) concentration in leaves and roots increased under NaCl salinity, whereas chloride (Cl^-^) concentration was higher in both the saline treatments and occurred in increasing pattern with Cl^-^ content in the external nutrient solution. The highest concentrations of Na^+^ and Cl^-^ were found in leaves, and in particular in those from NaCl and CaCl_2_ treated plants, respectively (Table 4). Under NaCl treatment, Na^+^ concentration in *C. citrinus* leaves increased by 6.1-fold compared to non-salt control, whereas under NaCl and CaCl_2_ stress treatments, Cl^-^ concentration in leaf tissue increased by 3.0 and 7.1-fold, respectively, compared to non-saline nutrient solution (Table 4). Moreover, under NaCl treatment, the decrease in K^+^/Na^+^ ratio in relation to the non-stressed control was higher in leaves than in roots, according to the higher levels of Na^+^ but not to the decrease in K^+^ concentration, which was not significant (Table 4).

**Table 4:** Effects of salt treatment in the nutrient solution on macronutrients, sodium, chloride and potassium-to-sodium ratio of leaves and roots of potted *Callistemon citrinus* and *Viburnum lucidum* plants.

	Ntot (g kg^-1^dw)		NO^3-^ (g kg^-1^dw)		PO_4_^3-^ (g kg^-1^dw)		K^+^ (g kg^-1^dw)		Ca^2+^ (g kg^-1^dw)		Mg^2+^ (g kg^-1^dw)		Na^+^ (g kg^-1^ dw)		Cl^-^ (g kg^-1^ dw)		K^+^/Na^+^	
*Callistemon citrinus*	leaves	roots	leaves	roots	leaves	roots	leaves	roots	leaves	roots	leaves	roots	leaves	roots	leaves	roots	leaves	roots
Salt treatment																		
Control	20.09 a	10.27	0.079 b	1.13 a	7.94 a	11.73 a	11.10 b	5.26 a	0.72 b	0.76 b	1.10 b	0.51	0.74 b	0.78 b	3.15 c	0.87 b	15.22 a	7.39 a
NaCl	18.56 b	10.67	0.078 b	0.81 b	4.49 b	9.65 ab	9.437 b	4.20 b	0.67 b	0.57 b	1.01 b	0.54	4.53 a	3.84 a	9.59 b	3.76 a	2.18 b	1.16 b
CaCl_2_	17.30 c	11.09	0.135 a	0.60 b	1.50 c	7.81 b	15.32 a	3.96 b	4.70 a	1.82 a	1.59 a	0.68	0.88 b	0.73 b	22.50 a	3.33 a	17.37 a	5.69 a
Significance	^∗∗∗^	NS	^∗^	^∗^	^∗∗∗^	^∗^	^∗∗∗^	^∗^	^∗∗∗^	^∗∗∗^	^∗∗∗^	NS	^∗∗∗^	^∗∗∗^	^∗∗∗^	^∗∗∗^	^∗∗∗^	^∗∗∗^

***Viburnum lucidum***	**leaves**	**roots**	**leaves**	**roots**	**leaves**	**roots**	**leaves**	**roots**	**leaves**	**roots**	**leaves**	**roots**	**leaves**	**roots**	**leaves**	**roots**	**leaves**	**roots**

Salt treatment																		
Control	25.39 a	13.79 a	3.67 a	7.59 a	10.58 a	7.33 a	28.06 a	11.4 a	1.81 b	1.31 b	1.42a	1.55 a	0.31 b	1.98 b	2.96 c	2.21 c	98.67 a	5.81 a
NaCl	24.00 a	12.31 b	0.94 b	2.63 b	8.09 b	3.59 b	19.81 b	4.65 c	0.91b	0.78 b	0.94 b	1.08 b	4.15 a	8.00 a	7.27 b	6.64 b	4.89c	0.58 b
CaCl_2_	21.89 b	12.45 b	0.33 b	1.30 b	4.90 b	2.86 b	20.07 b	8.60 b	7.19 a	2.49 a	1.63 a	1.33 b	0.63 b	1.48 b	13.25 a	9.13 a	46.02 b	5.98 a
Significance	^∗∗^	^∗^	^∗∗∗^	^∗∗∗^	^∗∗^	^∗∗∗^	^∗∗^	^∗∗∗^	^∗∗∗^	^∗∗∗^	^∗∗∗^	^∗∗^	^∗∗∗^	^∗∗∗^	^∗∗∗^	^∗∗∗^	^∗∗∗^	^∗∗∗^

*NS, ^∗^; ^∗∗^, ^∗∗∗^Non significant or significant at *P* < 0.05, 0.01, and 0.001, respectively. Different letters within each column indicate significant differences according to Duncan’s multiple-range test (*P* ≤ 0.05).*

In *V. lucidum*, the N and PO_4_^3-^ concentrations in leaves were more negatively influenced by CaCl_2_ than by NaCl (Table 4). The application of 53.33 mM CaCl_2_ in the nutrient solution positively affected the Ca^2+^ concentration in both leaves and roots; and in particular, in leaves it was 3.97 and 7.9-fold higher than that of non-salinized and NaCl^-^treated plants, respectively (Table 4). Similarly, to *C. citrinus*, the toxic ion (Na^+^ and Cl^-^) concentrations in leaves and roots occurred in increasing pattern with increasing external NaCl and CaCl_2_ stress (Table 4). Under NaCl conditions, Na^+^ and Cl^-^ concentrations in *V. lucidum* leaves and roots increased by 13.4/2.5-fold (for leaves) and 4.0/3.0-fold (for roots), respectively, compared to non-salt control (Table 4). Our results also showed that CaCl_2_ elicited a significant increase in Cl^-^ concentration in both leaves and roots, 4.5 and 4.1-fold, respectively, compared to non-saline treatment (Table 4). Finally, the decrease in K^+^/Na^+^ ratio under NaCl conditions in relation to the non-stressed control was higher in leaves than in and roots (Table 4).

### Leaf Functional Anatomical Traits

In *C. citrinus* leaves, the stomatal frequency (SF), guard cells length (GCL) and TLL were significantly affected by salinity sources (Table 5). More specifically, SF increased by 13% under NaCl, while GCL was decreased by NaCl treatment (by 12.3%) and increased by CaCl_2_ one (by 6%) (Table 5). TLL was decreased by both salinity treatments (Table 5). Moreover, no significant differences among treatments were observed for the incidence of both palisade and spongy parenchymas (TPP/TLL and TSP/TLL) over the total leaf lamina (Table 5).

**Table 5:** Effects of salt treatment in the nutrient solution on stomatal frequency (SF), guard cell length (GCL), thickness of leaf lamina (TLL), thickness of palisade parenchyma (TPP)/thickness of leaf blade (TLB) ratio, thickness of spongy parenchyma (TSP)/thickness of leaf blade (TLB) ratio and quantity of intercellular spaces in the spongy parenchyma (ISS) of potted *Callistemon citrinus* and *Viburnum lucidum* plants.

	SF	GCL	TLL	TPP/	TSP/	ISS
	(n. mm^-2^)	(μm)	(μm)	TLB	TLB	(%)
***Callistemon citrinus***						
Salt treatment						
Control	168.44 b	25.48 b	264.10 a	0.393	0.487	n.d.
NaCl	190.75 a	22.34 c	247.34 b	0.382	0.479	n.d.
CaCl_2_	152.57 b	27.14 a	252.40 b	0.386	0.482	n.d.
Significance	^∗∗∗^	^∗∗∗^	^∗∗^	NS	NS	
***Viburnum lucidum***						
Salt treatment						
Control	94.06	34.21 a	281.95 a	0.303	0.628 a	0.949 a
NaCl	99.55	29.25 b	244.61 b	0.335	0.581 b	0.888 b
CaCl_2_	95.46	28.90 b	235.46 b	0.318	0.599 b	0.896 b
Significance	NS	^∗∗∗^	^∗∗∗^	NS	^∗∗^	^∗∗∗^

*NS, ^∗∗^, ^∗∗∗^Non significant or significant at *P* < 0.01, and 0.001, respectively. Different letters within each column indicate significant differences according to Duncan’s multiple-range test (*P* ≤ 0.05). n.d. not detected.*

In *V. lucidum* leaves, the GCL, TLL, incidence of spongy parenchyma over the total leaf lamina (TSP/TLL) as well as the quantity of intercellular spaces in the spongy parenchyma (ISS) were significantly affected by salinity sources (Table 5). The application of 80 mM NaCl or 53.33 mM CaCl_2_ in the nutrient solution determined a significant decrease in GCL, TLL, TSP/TLB and ISS (by 15.0, 14.9, 6.0, and 6.0%, respectively) with no significant difference between the two salinity sources (Table 5). Finally, no significant differences among salt treatments were observed for both SF (avg. 96.4 n. mm^-2^) and incidence of palisade parenchyma over the total leaf lamina TPP/TLL (avg. 0.319) (Table 5).

### Heat Map Analysis

**FIGURE 1:**
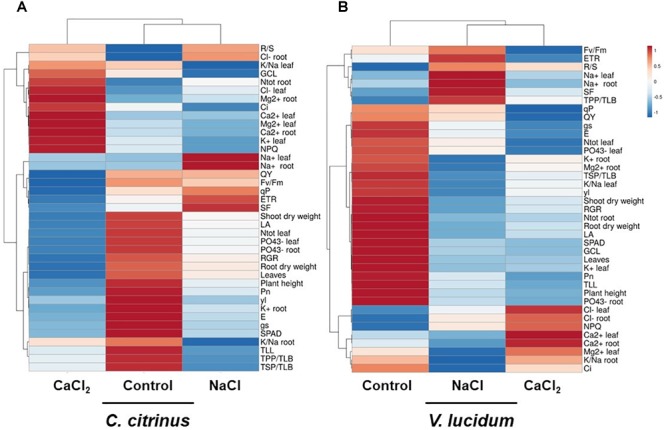
Cluster heat map analysis summarizing potted *Callistemon citrinus* **(A)** and *Viburnum lucidum* **(B)** responses to non-saline, NaCl and CaCl_2_ salinity treatments (performed in terms of equimolar concentrations). The heat map was generated using the https://biit.cs.ut.ee/clustvis/ online program package with Euclidean distance as the similarity measure and hierarchical clustering with complete linkage.

A heat map providing the morpho-anatomical, biochemical and physiological changes of potted *C. citrinus* and *V. lucidum* in response to salinity sources (non-saline, NaCl or CaCl_2_) was displayed in Figure 1. The heat-map identified two main clusters in both ornamental species, which, however, divided the analyzed samples differently (Figure 1). For instance, in *C. citrinus* CaCl_2_ was completely separated from control and NaCl treatments (Figure 1A), while in *V. lucidum* the non-saline control was clearly separated from the two salinity sources (Figure 1B). Our results indicated that while in *C. citrinus* Cl^-^ and Ca^2+^ salinity in both leaves and roots were the main clustering factor, followed by Na^+^; in *V. lucidum* the negative effect of salinity, even depending on different ions, Na^+^ for NaCl salt stress and Cl^-^ and Ca^2+^ for CaCl_2_, induced similar negative effects on plant morphological and physiological parameters compared to the non-saline treatment. In particular, CaCl_2_ treatment clustered separated from the other two treatments in *C. citrinus* because of its higher sub-stomatal CO_2_ concentration (C_i_), NPQ, total N in the root, Cl^-^ and K^+^ in leaves, the higher Ca^2+^ and Mg^2+^ concentrations in both dried tissues, as well as its lower QY, F_v_/F_m_, ETR, root dry weight, RGR and number of leaves compared to the other two treatments (Figure 1A). On the other hand, the two equimolar concentrations of NaCl and CaCl_2_ clustered separated from control treatment in *V. lucidum* mainly due to their lower K^+^ and PO_4_^3-^ concentrations in roots and leaves, as well as the lower photosynthetic performance (Pn), RGR, plant height, number of leaves, root and shoot dry weight (Figure 1B).

### Principal Component Analysis

**FIGURE 2:**
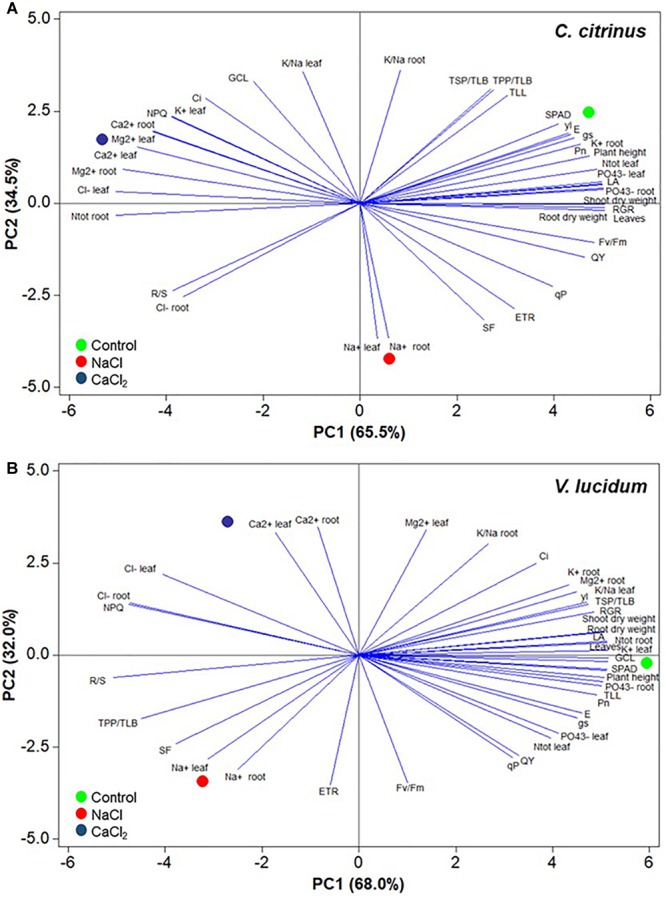
Principal component loading plot and scores of principal component analysis (PCA) of morpho-anatomical, biochemical, physiological parameters and ion contents and partitioning of potted *Callistemon citrinus* **(A)** and *Viburnum lucidum* **(B)** grown under non-saline, NaCl and CaCl_2_ salinity treatments.

The principal component analysis (PCA) showed that for both species, the first two principal components (PCs) were associated with Eigen values higher than one and explained 100% of the cumulative variance, with PC1 and PC2 accounting for 65.5 and 34.5% for *C. citrinus* (Figure 2A) and 68.0 and 32.0% for *V. lucidum* (Figure 2B).

In *C. citrinus* PC1 was positively correlated to RGR, number of leaves, root and shoot dry weight, leaf area, PO_4_^3-^ and N total concentrations in leaves, and also with PO_4_^3-^ and K^+^ in roots, QY, Pn, plant height, F_v_/F_m_ and other photosynthetic parameters. PC1 was also negatively correlated to leaf Cl^-^, Ca^2+^ Mg^2+^ and K^+^ concentrations, as well as root N, Ca^2+^ and Mg^2+^ contents, NPQ and R/S ratio. Furthermore, PC2 was positively correlated to K^+^/Na^+^ in both plant tissues, GCL,TSP/TLB, TPP/TLB, TLL, and Ci, and negatively correlated to Na^+^ content in leaves and Na^+^ and Cl^-^ content in roots, SF, ETR and R/S (Figure 2A). In *V. lucidum* PC1 was positively correlated to K^+^ in leaves, number of leaves, GCL, SPAD, N, and PO_4_^3-^ in roots, LA, root and shoot dry weight, plant height, TLL, RGR and Pn. PC1 was also negatively correlated to R/S ratio, NPQ, leaf and root TPP/TLB and SF. Finally, PC2 was positively correlated to root and leaf Ca^2+^, leaf Mg^2+^ content, K^+^/Na^+^ in roots and Ci, TSP/TLB, and negatively correlated to ETR, Fv/Fm, Na^+^ concentration in both organs, qP, QY and SF (Figure 2B).

In the current experiment, the score plot of the PCA superimposed on the above matrix of variables in both species revealed strong clustering of the three nutrient solutions, with *C. citrinus* treated with non-saline solution concentrating plant growth parameters, SPAD index, mineral status (N, P, K), photosynthesis activity, TPP/TLB, TSP/TLB, TLL, whereas the *V. lucidum* treated with non-salinized solution stands out for plant growth parameters, most mineral composition and physiological parameters. Particularly, the *C. citrinus* and *V. lucidum* under control treatment were positioned on the positive side of PC1 in the upper (for *C. citrinus*) and between the higher and lower right quadrants of the PCA score plot (for *V. lucidum*; Figure 2).

The lower right quadrant included *C. citrinus* treated with NaCl that delivered leaves and roots with high concentration of sodium, whereas in the upper left quadrant was positioned *C. citrinus* treated with CaCl_2_ characterized by high concentration of monovalent and bivalent cations and NPQ but also high chloride concentration (Figure 2). Finally, in *V. lucidum* the upper left quadrant depicted CaCl_2_ treatment characterized by high levels of calcium and chloride as well as high NPQ value, whereas the lower left quadrant (NaCl treatment) stands out for high leaf and root sodium concentrations and high values of R/S ratio, TPP/TLB, SF, and ETR (Figure 2).

## Discussion

Even though many studies have been carried out on the effects of water salinity on growth performances and tolerance responses of ornamental shrubs (Álvarez and Sánchez-Blanco, 2014; Acosta-Motos et al., 2015; García-Caparrós and Lao, 2018 and therein literature), little information is available on their adaptive mechanisms to cope with different salt stress sources.

The two ornamental shrubs, *C. citrinus* and *V. lucidum*, showed different responses to salt treatments concerning growth, anatomical functional traits and photosynthesis. The reduction in plant height and shoot dry weight recorded in the analyzed species under 80 mM NaCl resulted slightly higher when compared to those reported by Álvarez and Sánchez-Blanco (2014) and Cassaniti et al. (2009) and lower compared to the results obtained when the two species were treated with 200 mM NaCl salinized water (Cirillo et al., 2016). In spite of the imposed iso-osmotic salinity of the compared treatments, the effect of 53.33 mM CaCl_2_ on the growth parameters was more marked in *C. citrinus* than in *V. lucidum*. To the best of our knowledge, there are no comparative studies on the responses of ornamental shrubs to iso-osmotic saline irrigation with NaCl and CaCl_2_, whereas only few reports on vegetable species are available on the CaCl_2_ salinity (Colla et al., 2012, 2013; Ntatsi et al., 2017; Rabhi et al., 2018). In particular, 40 mM CaCl_2_ significantly reduced the root, stem and leaf dry weight of saffron plants compared to control (Rabhi et al., 2018), while in sunflower plants, saline irrigation with NaCl or CaCl_2_ at EC of 5.0 dS m^-1^ induced a similar decline in flower size and biomass (de Sousa Júnior et al., 2017). The two ornamental species differently responded to the salinity treatments in the ratio between below- and above-ground biomass. Indeed, the increase in root to shoot ratio is a frequently detectable response to salt stress, more related to the osmotic effect than to a salt-specific effect (Hsiao and Xu, 2000). In *V. lucidum* the marked increase in root to shoot ratio, possibly favoring the retention of toxic Na^+^ ions at the root level, may be associated with lower salt tolerance (Dalton et al., 1997). In fact, *V. lucidum*, retained Na^+^ in roots at a higher extent than in the leaves; while *C. citrinus* had a higher ability to retain Na^+^ and Cl^-^ levels in leaves than in roots under NaCl and CaCl_2_ salinity, respectively. This suggests the existence of an inclusion mechanism similar to that adopted by other Mediterranean ornamental plants under salinity (Navarro et al., 2008; Álvarez et al., 2012; Carillo et al., 2019), which is considered as a trait of salt tolerance that confers plants adaptive plasticity to osmotic and ionic stress (Läuchli and Epstein, 1990; Rodríguez et al., 2005; Woodrow et al., 2011). Indeed, the compartmentalization of toxic ions as cheap osmotica in the vacuole and the synthesis and accumulation of osmolytes in the cytosol are essential mechanisms of osmotic adjustment and oxidative stress protection in plant cells (Carillo, 2018; Ferchichi et al., 2018; Annunziata et al., 2019).

Acosta-Motos et al. (2017) reported that *C. citrinus* behaves as a typical Cl^-^ accumulator, because it preferentially partitions Cl^-^ into vacuoles. The ability of *C. citrinus* in compartmentalizing high concentration of Cl^-^ in vacuoles allowed also these plants to avoid the competition of Cl^-^ with NO_3_^-^ for translocation within the plants by NO_3_^-^ transporter proteins, leading to concentrations of NO_3_^-^ in leaves which were unchanged or double compared to that of the non-saline control in NaCl and CaCl_2_ treatments, respectively, while, in *V. lucidum* leaves, the concentration of this ion underwent a strong decrease compared to the control. Notwithstanding the higher ability of *C. citrinus* to retain excess Cl^-^ in the leaves, even if for the most part compartmentalized in the vacuole, it still caused in this plant the decrease in both PSII quantum yield and photosynthetic electron transport rate, usual symptoms of Cl^-^ toxicity (Tavakkoli et al., 2010).

In *V. lucidum* the increase in uptake and transport of Na^+^ under NaCl salinity strongly interfered with K^+^ uptake in roots at the plasma membrane, even decreasing K^+^ transport to leaves (Gao et al., 2016), with a consequent strong reduction of K^+^ to Na^+^ ratio, a far more important parameter than the absolute amount of Na^+^ (Shabala and Munns, 2012). High Na^+^ concentrations are able to depolarize and damage the plasma membrane causing a restriction of K^+^ uptake and leaking; besides, Na^+^ can substitute K^+^ in key enzymatic reactions, further impairing cellular functions in cytosol and organelles (Almeida et al., 2017; Carillo et al., 2019). In addition, excess Ca^2+^, unlikely causing toxicity in itself, can restrain the uptake or availability of other nutrients such as K^+^ in *V. lucidum*, or PO_4_^3-^ in *C. citrinus* contributing to restrain RGR (White and Broadley, 2003).

Plants try to allocate Ca^2+^ and PO_4_^3-^ to different cell types in order to avoid the formation and precipitation of Ca_3_(PO_4_)_2_ crystals and render Ca^2+^ or PO_4_^3-^ unavailable for metabolism and growth (Conn and Gilliham, 2010; Ding et al., 2018). However, when plants are grown under CaCl_2_ salinity, Ca^2+^ is present at excessive concentrations in almost all root and leaf tissues, and therefore, interacting with PO_4_^3-^ or even oxalate, it is accumulated in crystals from which it will be released only when Ca^2+^ levels will decrease, in order to buffer its biologically active levels in plants (Nazrul Islam and Kawasaki, 2015). Thus, the concentrations of available PO_4_^3-^ tended to strongly decrease in *C. citrinus* leaves where the Ca^2+^ concentration showed the highest increase compared to the respective control.

Greenway and Munns (1980) reported that even if Na^+^ concentration in plant tissues increases under salinity, not necessarily the K^+^ to Na^+^ ratio in the cytoplasm is drastically reduced. *C. citrinus* has certainly the ability to retain cytosolic K^+^ concentration at a constant level or even increased by using the K^+^ stored in the vacuole compared to *V. lucidum*. The capability to satisfy plant metabolic demand for K^+^ under salinity by compartmentalizing Na^+^ in the vacuole and the majority of K^+^ in the cytosol could be another essential mechanism for *C. citrinus* salt tolerance (Wang et al., 2013). In fact, the K^+^ accumulated in the cytosol could also contribute to the osmotic balance of the toxic ions compartmentalized in the vacuole. In fact, even low K^+^ concentrations, if compartmentalized in the cytosol which usually occupies less than 10% of cell volume, can be sufficient to determine a significant osmotic pressure able to balance the vacuolar osmotic potential (Cuin et al., 2009).

The severe ion imbalance and physiological disorders related to salinity may also affect leaf gas exchange and plant growth (García-Caparrós and Lao, 2018). Based on our data, dark reactions of photosynthesis might be supposed more sensitive than light reactions to different salt treatments: gas exchanges evidenced a significant decline in net photosynthesis, stomatal conductance and transpiration for both species under NaCl as well as CaCl_2_ treatments compared to unstressed controls accompanied by the reduction of SPAD index and leaf area.

In several ornamental shrubs, NaCl salinity decreased net assimilation rate through stomatal and/or non stomatal limitations, such as the unbalancing of the electron transport chain and/or the impairment of the Calvin Cycle enzymes (Chaves et al., 2009; Álvarez et al., 2012; Álvarez and Sánchez-Blanco, 2014; Cirillo et al., 2016). Consistently with the data obtained for plant growth, in *C. citrinus* plants, NaCl lowered Pn rate less than CaCl_2_. The stronger decline of photosynthesis observed under CaCl_2_ treatment was not accompanied by an equally strong reduction in stomatal conductance. This latter, occurring at the same extent in both the saline treatments was not able to explain alone the dramatic reduction in the leaf photosynthetic rate.

The lower photosynthesis induced by salt treatments may be also a direct consequence of the salt-induced reduction of leaf lamina thickness: indeed, the area-based photosynthetic capacity is directly proportional to leaf thickness (Wyka et al., 2012). However, while *C. citrinus* plants exposed to NaCl salinity may partly compensate such a reduction in potential photosynthetic capacity thanks to the occurrence of more frequent but smaller stomata; the lack of such a structural adjustment in CaCl_2_-treated plants would support their more severe decline in photosynthesis. Indeed, the occurrence of more frequent but smaller stomata, exerting a better control of stomatal aperture, has been recorded also in other ornamental species subjected to salinity stress (Carillo et al., 2019), and is recognized as a structural adjustment to achieve a better control of gas exchanges (Raven, 2014).

On the contrary, in *V. lucidum* salt treated plants responded to both the salt sources with a similar reduction of stomatal conductance and Pn rate. Such common tendency of variation in *V lucidum* plants in response to the two salt types is maintained also in structural adjustments. *V. lucidum* NaCl- and CaCl_2_- treated plants behave as *C. citrinum* CaCl_2_-treated plants, developing thinner leaves with smaller, but not more frequent, stomata. The occurrence of thinner lamina under both salt treatments is not due to a reduction in the thickness of the palisade parenchyma, thus indicating the maintenance of the main tissue devoted to photosynthesis. However, the occurrence of less intercellular spaces at the spongy tissue level would also indicate the occurrence of changes in the mesophyll resistance (Sack and Frole, 2006). The increase in the amount of intercellular spaces has often been linked with the ability to cope with salinity by improving the CO_2_ diffusion in the mesophyll, thus compensating for salinity-induced increased stomatal limitations (Acosta-Motos et al., 2015; Rouphael et al., 2017c; Carillo, 2018). In *V. lucidum*, the decrease in intercellular spaces would indicate a different strategy pointing at increasing water use efficiency in terms of carbon fixed per water lost through transpiration (McAusland et al., 2016).

The slightest effects exerted by NaCl on the photochemistry of *C. citrinus*, in particular related to reduction in stomatal conductance or injuries to the photosynthetic apparatus, compared to CaCl_2_ were probably due to a direct effect of salt stress and were consistent with the lowest RGR (Rodríguez et al., 2005; Moradi and Ismail, 2007). In fact, in *C. citrinus* plants grown under CaCl_2_, conversely to plants treated with NaCl, the internal CO_2_ concentration was increased in sub-stomatal cavities (Ci of NaCl treated plants does not differ compared to control). This supported the hypothesis that, beside stomatal closure, the stronger photosynthetic decline associated with the addition of CaCl_2_ in the nutritional solution was also due to possible biochemical limitations, as indicated by the reduction in the electron transport rate. On the other hand, *V. lucidum* plants treated with both salts, exhibited a similar decline in stomatal conductance and transpiration but no changes in internal CO_2_ concentration. However, in both species, the efficiency of photosystem II in light harvesting and conversion declined under CaCl_2_ treatment as well as the electron transport rate, suggesting a simultaneous corruption of reductive power and proton gradient generation (for the synthesis of NADPH and ATP) in the light reactions of photosynthesis. This kind of damage might be due to the cytotoxic effect of the increased concentration of Cl^-^ ions in the photosynthetic tissues (Tavakkoli et al., 2010, 2011). At the same time, in these plants, the excess of absorbed light not utilized in photochemistry was dissipated as heat as indicated by the rising non-photochemical quenching (NPQ) in response to the CaCl_2_ treatment. Under salt stress, as well as under unfavorable environmental conditions, the thermal dissipation within photosystems acts as a safe strategy for cutting down the surplus of light energy and minimizing ROS generation (Azzabi et al., 2012). Nevertheless, such a compensatory mechanism was not able to prevent photoinhibition, as suggested by the significant reduction of F_v_/F_m_ in CaCl_2_ plants of both species compared to the respective control. A possible reason for the photochemical drop in CaCl_2_ plants may be related to the observed concurrent reduction of SPAD index and leaf area in these leaves, indicating, respectively, an impairment of the antenna system in light harvesting and/or a limitation in light absorption due to the reduced leaf lamina size, as previously mentioned. Besides, it cannot be excluded that salinity might have induced oxidative stress at the subcellular level, mainly in the chloroplasts, affecting the whole photosynthetic process.

## Conclusion

The results of this study allowed discriminating the effects of specific osmotic stress and ion toxicity on *Callistemon citrinus* and *Viburnum lucidum* ornamental shrubs, assessing how they activated differential responses to a similar osmotic stress but induced by distinct salinity sources. Both the shrub species displayed multiple adaptive mechanisms to counteract harmful salinity effects, confirming their low sensitivity to a rather high threshold of salt concentration in the irrigation water imposed for a moderately long period. In particular, shoot dry weight, leaf number, total leaf area and net photosynthetic rate were similarly, restrained in both *C. citrinus* and *V. lucidum* under NaCl, whereas the two species exhibited a different response to CaCl_2_ salinity. *C. citrinus* plants exposed to NaCl salinity may partly compensate such a reduction in potential photosynthetic capacity thanks to the occurrence of more frequent but smaller stomata. On the contrary, *V. lucidum* responded to both the salt sources, as *C. citrinum* under CaCl_2_, developing thinner leaves with smaller, but not more frequent stomata. However, the strong drop in photosynthetic rate of *C. citrinus* induced by CaCl_2_, likely ascribed to both stomatal and non stomatal limitations, was more severe than that of the other plant and/or other treatment and determined a stronger growth reduction. In fact, in this species the reduction in photosynthesis occurred together with the decline in PSII photochemistry, and the increase in non-photochemical dissipation processes. However, these plants displayed an increased ability to retain higher Cl^-^ levels in leaves than in roots under CaCl_2_ salinity compared to *V. lucidum*, thus, indicating a further attempt to counteract Cl^-^ toxic effects through its increased vacuolar compartmentalization and use as cheap osmoticum. The results of this study may provide useful indications in the selection of shrubs suitable for the urban, industrial and marginal areas’ xeriscaping, when resorting to reclaimed water for plant irrigation may represent a compelling option to water scarcity.

## Author Contributions

All authors listed have made a substantial, direct and intellectual contribution to the work, and approved it for publication.

## Conflict of Interest Statement

The authors declare that the research was conducted in the absence of any commercial or financial relationships that could be construed as a potential conflict of interest. The reviewer GF declared a past co-authorship with several of the authors CC, AP, SP, and YR to the handling Editor.

## Footnotes

1 www.ibm.com/software/analytics/spss
